# 

**Published:** 1895-12

**Authors:** 


### CONTENTS.

### *EDITORIAL.*

PAGE

Arsenicum-album, . . . . . . . . . . 537

### *MISCELLANEOUS.*

Repertory of the Symptoms of the Ovaries. C. M. Boger, M. D., . . . . . . . . . . 540
The Ideal Sanitation of a Physician's Office. Bushrod W. James, A. M., M. D., . . 548
*The Organon* and Materia Medica Club of the Bay Cities of California, . . . . . . . 552
Dr. Pease's Case Reported in the October Number, . . . . . . . . . . 561
A Remarkable Case of Abstinence, . . . . . . . . . . 562
Symptoms from Morphine, . . . . . . . . . . 563
Vomiting of Fluids, but not of Solids, . . . . . . . . . . 563
In Memoriam—Dr. Guy A. T. Lincoln, . . . . . . . . . . 565
Tendon Grafting. Samuel E. Milliken, M. D., . . . . . . . . . . 565
Carleton's Genito-Urinary and Veneral Diseases. Bœricke, Runyon and Ernesty, . 566
Headache of Several Years' Standing Cured by Natrum-muriaticum. Joseph T. O'Connor, M. D., . . . . . . . . . . 567
Hypericum for Convulsions. A. S. Ironside, M. D., . . . . . . . . . . 568
"It Do Move," . . . . . . . . . . 568
Reminiscences of Dr. Mahlon Preston, . . . . . . . . . . 569

### *BOOK NOTICES.*

Materia Medica and Therapeutics, . . . . . . . . . . 570
Practical Uranalysis and Urinary Diagnosis, . . . . . . . . . . 571
Essentials of Homœopathic Therapeutics, . . . . . . . . . . 572
Physician's Visiting List for 1896, . . . . . . . . . . 573
Climate and Health, . . . . . . . . . . 573
Transactions of the American Institute of Homœopathy, . . . . . . . . . . 573
Transactions of the New York State Society . . . . . . . . . . 574
The Scientific American, . . . . . . . . . . 574
The International Medical Annual for 1896, . . . . . . . . . . 574

### *NOTES AND NOTICES.*

Lippe.—Dunham Medical College.—How we intend to Check Substitution of Drugs, 575

